# The genome sequence of the peacock moth, *Macaria notata* (Linnaeus, 1758)

**DOI:** 10.12688/wellcomeopenres.18108.1

**Published:** 2022-09-12

**Authors:** Douglas Boyes, University of Oxford and Wytham Woods Genome Acquisition Lab, Darwin Tree of Life Barcoding collective, Wellcome Sanger Institute Tree of Life programme, Wellcome Sanger Institute Scientific Operations: DNA Pipelines collective, Tree of Life Core Informatics collective, Christian Couch, David Plotkin, Akito Y. Kawahara, Darwin Tree of Life Consortium

**Affiliations:** 1 UK Centre for Ecology and Hydrology, Wallingford, Oxfordshire, UK; 2 McGuire Center for Lepidoptera and Biodiversity, Florida Museum of Natural History, Gainesville, Florida, USA

**Keywords:** Macaria notata, peacock moth, genome sequence, chromosomal, Geometridae

## Abstract

We present a genome assembly from an individual male *Macaria notata* (the peacock moth; Arthropoda; Insecta; Lepidoptera; Geometridae). The genome sequence is 394 megabases in span. The majority of the assembly (99.98%) is scaffolded into 29 chromosomal pseudomolecules with the Z sex chromosome assembled. The complete mitochondrial genome was also assembled and is 15.4 kilobases in length.

## Species taxonomy

Eukaryota; Metazoa; Ecdysozoa; Arthropoda; Hexapoda; Insecta; Pterygota; Neoptera; Endopterygota; Lepidoptera; Glossata; Ditrysia; Geometroidea; Geometridae; Ennominae; *Macaria*; *Macaria notata* (Linnaeus, 1758) (NCBI:txid688445).

## Background

The peacock moth, *Macaria notata* (Linnaeus, 1758) is a moth in the family Geometridae. This species has wings with a greyish-white ground colour dorsally, and whiter coloration with many small brown spots and stripes on the underside. The dorsal forewing has some brown patches along the costal margin, and a darker-brown patch covering part of the postmedial line. The forewing also has a notable brown, crescent-shaped concavity just below the apex. The hindwing margin is slightly elongated at the end of vein M _3 _( Friedrich, 2022).

This species thrives in Holarctic deciduous forests and meadows rich in thick brush. Caterpillars tend to feed on leaves of deciduous trees such as oak, birch, alder, poplar, and blackthorn ( Friedrich, 2022). The peacock moth’s Palearctic distribution ranges from central and northern Europe to eastern Siberia and Japan, and as far south as Iran. There are also populations in Canada and both the east and west coasts of the northern United States ( Moth Photographers Group, 2022). Its flight season ranges from May to September, with two generations in central Europe ( Friedrich, 2022). The moth is often attracted to light and can be collected commonly. Genomic research for this species could be used to determine zoogeographical patterns within multiple European states, and its relationship to other closely related species ( Can *et al.*, 2018; Õunap *et al.*, 2011) .

## Genome sequence report

**Figure 1.**
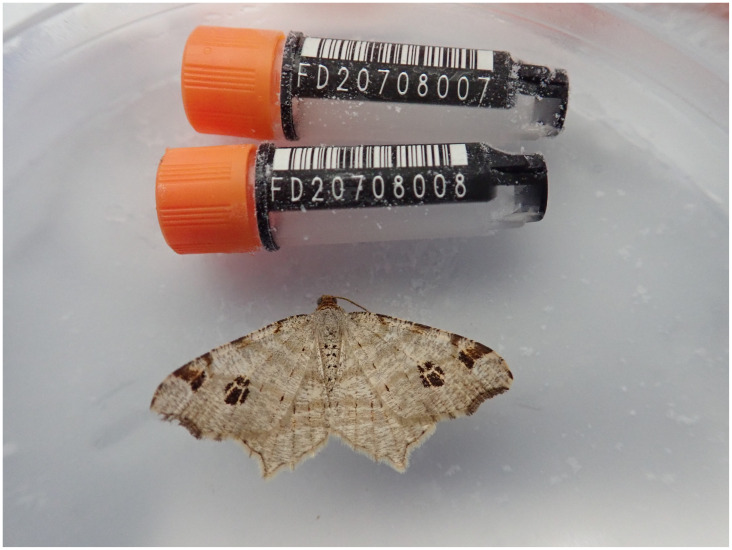
Image of the *Macaria notata* specimen taken prior to preservation and processing.

The genome was sequenced from a single male *M. notata* collected from Wytham Woods, Berkshire, UK ( Figure 1). A total of 58-fold coverage in Pacific Biosciences single-molecule HiFi long reads and 104-fold coverage in 10X Genomics read clouds were generated. Primary assembly contigs were scaffolded with chromosome conformation Hi-C data. Manual assembly curation corrected 1 misjoin, reducing the scaffold number by 3.13%.

**Figure 2.**
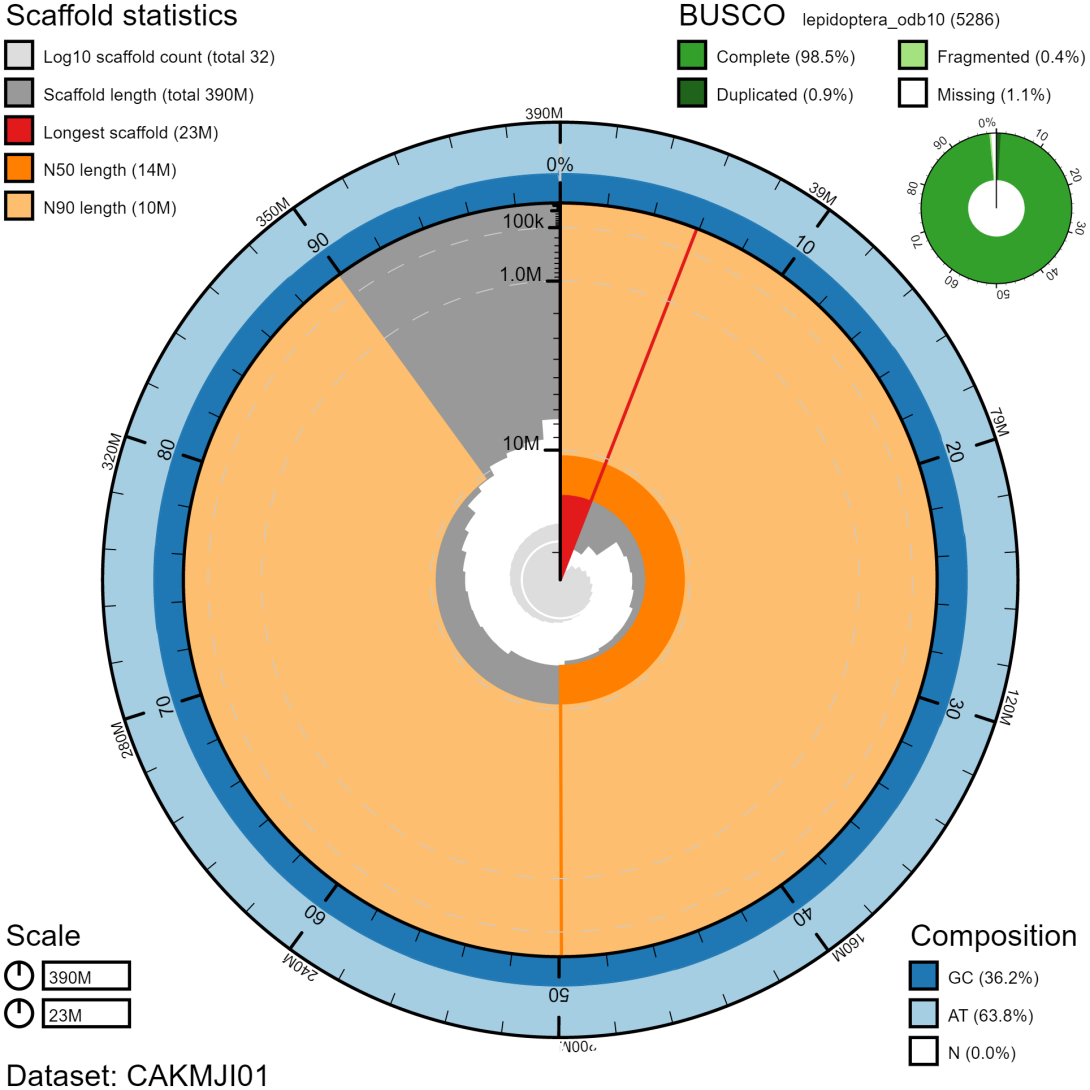
Genome assembly of *Macaria notata*, ilMacNota1.1: metrics. The BlobToolKit Snailplot shows N50 metrics and BUSCO gene completeness. The main plot is divided into 1,000 size-ordered bins around the circumference with each bin representing 0.1% of the 393,787,475 bp assembly. The distribution of chromosome lengths is shown in dark grey with the plot radius scaled to the longest chromosome present in the assembly (23,248,439 bp, shown in red). Orange and pale-orange arcs show the N50 and N90 chromosome lengths (13,783,192 and 10,416,434 bp), respectively. The pale grey spiral shows the cumulative chromosome count on a log scale with white scale lines showing successive orders of magnitude. The blue and pale-blue area around the outside of the plot shows the distribution of GC, AT and N percentages in the same bins as the inner plot. A summary of complete, fragmented, duplicated and missing BUSCO genes in the lepidoptera_odb10 set is shown in the top right. An interactive version of this figure is available at https://blobtoolkit.genomehubs.org/view/ilMacNota1.1/dataset/CAKMJI01/snail.

The final assembly has a total length of 394 Mb in 31 sequence scaffolds with a scaffold N50 of 13.8 Mb ( Table 1). The majority, 99.98%, of the assembly sequence was assigned to 29 chromosomal-level scaffolds, representing 28 autosomes (numbered by sequence length) and the Z sex chromosome ( Figure 2– Figure 5; Table 2).

**Table 1.** Genome data for *Macaria notata*, ilMacNota1.1.

*Project accession data*	
Assembly identifier	ilMacNota1.1
Species	*Macaria notata*
Specimen	ilMacNota1 (genome assembly, Hi-C)
NCBI taxonomy ID	688445
BioProject	PRJEB48591
BioSample ID	SAMEA7746623
Isolate information	Male. Abdomen tissue (genome assembly); head/thorax tissue (Hi-C)
*Raw data accessions*	
PacificBiosciences SEQUEL II	ERR7254658
10X Genomics Illumina	ERR7253245-ERR7253248
Hi-C Illumina	ERR7253249
*Genome assembly*	
Assembly accession	GCA_927399415.1
	GCA_927399385.1
Span (Mb)	394
Number of contigs	33
Contig N50 length (Mb)	13.8
Number of scaffolds	31
Scaffold N50 length (Mb)	13.8
Longest scaffold (Mb)	19.3
BUSCO * genome score	C:98.5%[S:97.6%,D:0.9%], F:0.4%,M:1.1%,n:5,286

*BUSCO scores based on the lepidoptera_odb10 BUSCO set using v5.3.2. C= complete [S= single copy, D=duplicated], F=fragmented, M=missing, n=number of orthologues in comparison. A full set of BUSCO scores is available at https://blobtoolkit.genomehubs.org/view/ilMacNota1.1/dataset/CAKMJI01/busco.

**Figure 3.**
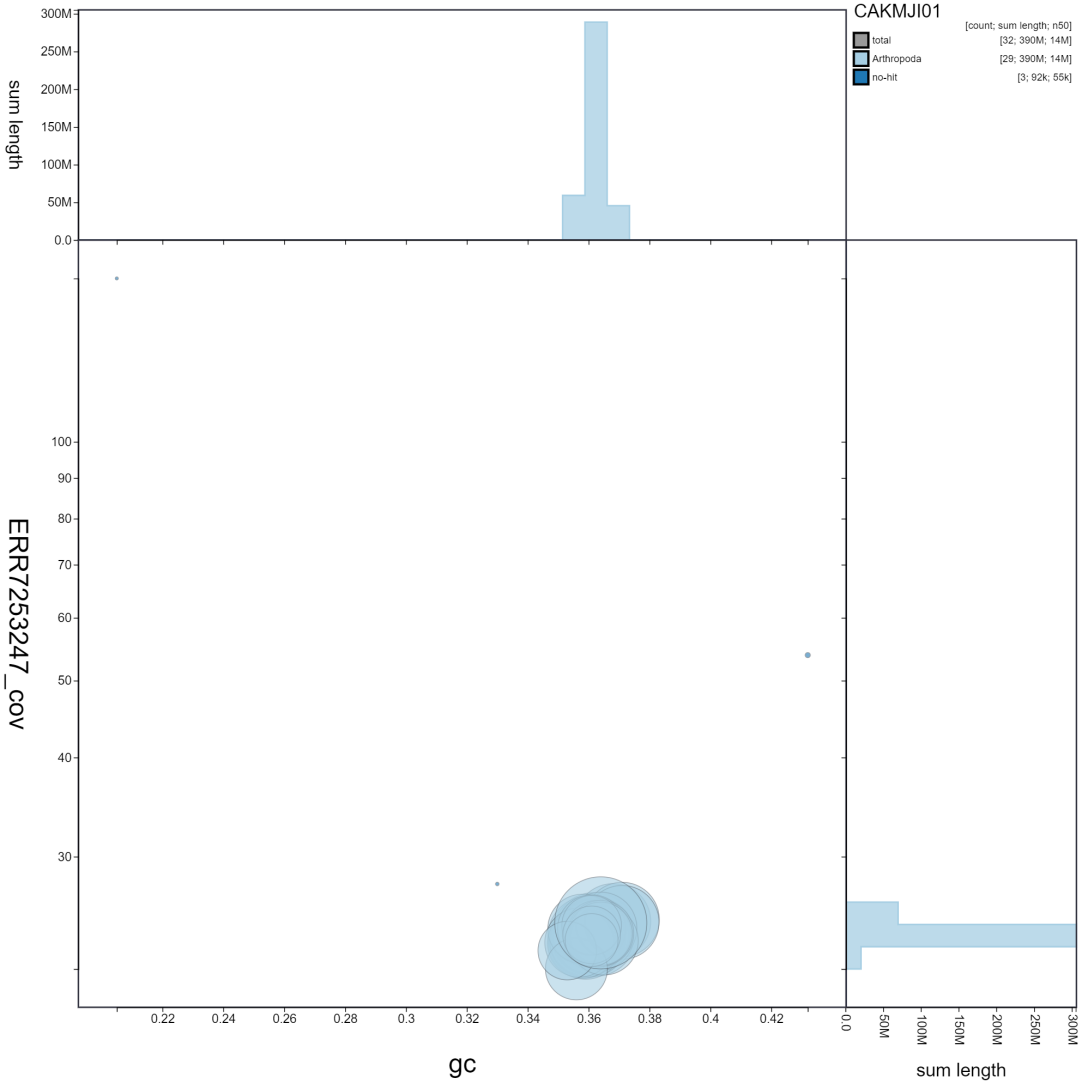
Genome assembly of *Macaria notata*, ilMacNota1.1: GC coverage. BlobToolKit GC-coverage plot. Scaffolds are coloured by phylum. Circles are sized in proportion to scaffold length. Histograms show the distribution of scaffold length sum along each axis. An interactive version of this figure is available at https://blobtoolkit.genomehubs.org/view/ilMacNota1.1/dataset/CAKMJI01/blob.

**Figure 4.**
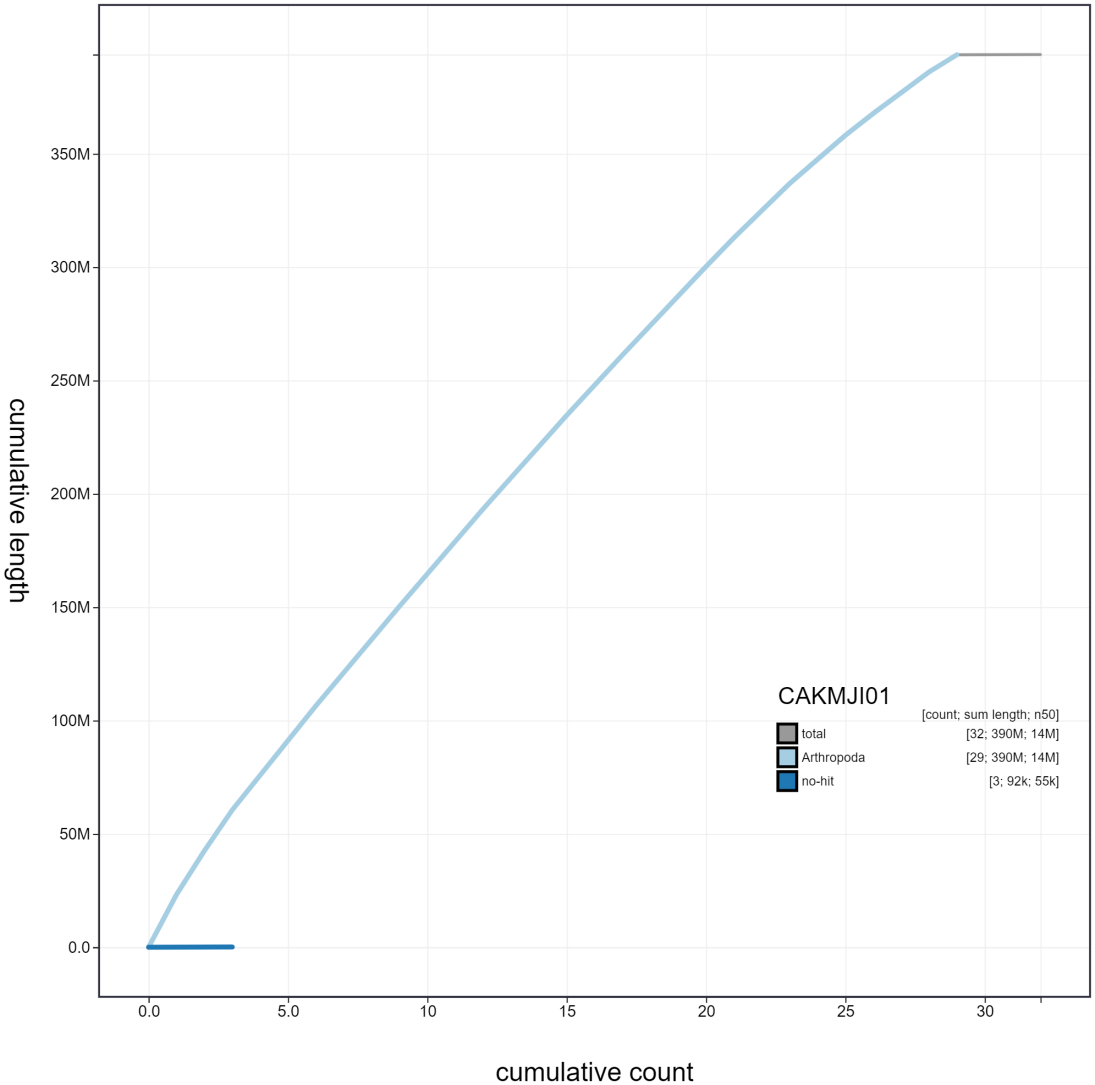
Genome assembly of *Macaria notata*, ilMacNota1.1: cumulative sequence. BlobToolKit cumulative sequence plot. The grey line shows cumulative length for all scaffolds. Coloured lines show cumulative lengths of scaffolds assigned to each phylum using the buscogenes taxrule. An interactive version of this figure is available at https://blobtoolkit.genomehubs.org/view/ilMacNota1.1/dataset/CAKMJI01/cumulative.

**Figure 5.**
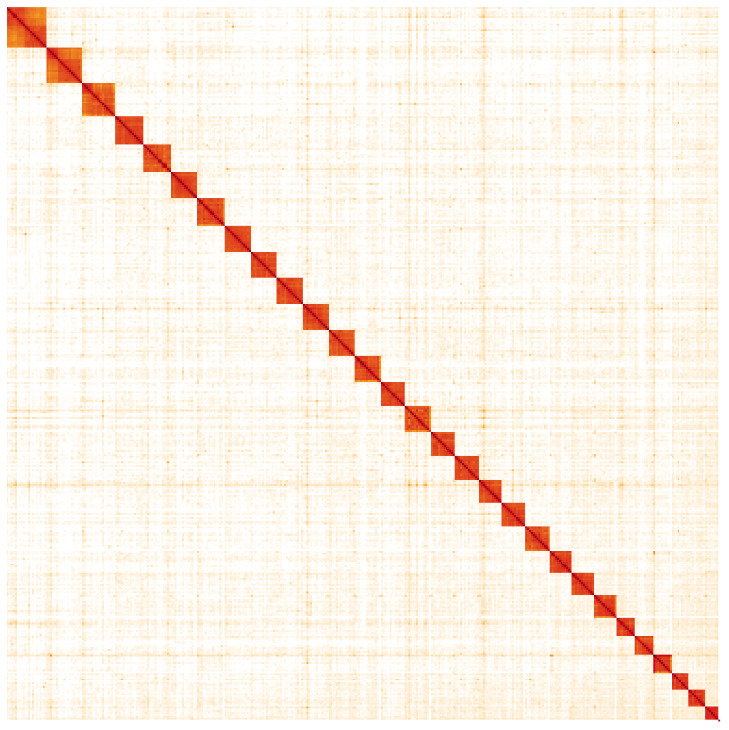
Genome assembly of *Macaria notata*, ilMacNota1.1: Hi-C contact map. Hi-C contact map of the ilMacNota1.1 assembly, visualised in HiGlass. Chromosomes are arranged in size order from left to right and top to bottom. The interactive Hi-C map can be viewed at https://genome-note-higlass.tol.sanger.ac.uk/l/?d=Ng_bHCybQ9aZiaw8aC3a4w.

**Table 2.** Chromosomal pseudomolecules in the genome assembly of *Macaria notata*, ilMacNota1.1.

INSDC accession	Chromosome	Size (Mb)	GC%
OV656725.1	1	19.26	36.2
OV656726.1	2	18.04	36
OV656727.1	3	15.55	36.8
OV656728.1	4	15.28	35.9
OV656729.1	5	15.11	37.1
OV656730.1	6	14.9	37.1
OV656731.1	7	14.52	36.3
OV656732.1	8	14.51	36
OV656733.1	9	14.45	36.2
OV656734.1	10	14.24	36.4
OV656735.1	11	14.22	35.9
OV656736.1	12	13.78	36.2
OV656737.1	13	13.78	36.2
OV656738.1	14	13.76	35.9
OV656739.1	15	13.31	36.2
OV656740.1	16	13.29	35.8
OV656741.1	17	13.13	35.8
OV656742.1	18	13.07	35.7
OV656743.1	19	12.89	36.5
OV656744.1	20	12.66	36.1
OV656745.1	21	12.18	36.3
OV656746.1	22	11.75	36.4
OV656747.1	23	10.85	36.4
OV656748.1	24	10.42	35.6
OV656749.1	25	9.63	36.1
OV656750.1	26	9.31	35.3
OV656751.1	27	9.01	36.1
OV656752.1	28	7.58	36.1
OV656724.1	Z	23.25	36.4
OV656753.1	MT	0.02	20.6
-	Unplaced	0.08	40.4

The assembly has a BUSCO v5.3.2 ( Manni *et al.*, 2021) completeness of 98.5% (single 97.6%, duplicated 0.9%) using the lepidoptera_odb10 reference set (n=5,286). While not fully phased, the assembly deposited is of one haplotype. Contigs corresponding to the second haplotype have also been deposited.

## Methods

### Sample acquisition and nucleic acid extraction

A single *M. notata* specimen (ilMacNota1) was collected using a light trap from Wytham Woods, Berkshire, UK (latitude 51.772, longitude -1.338) by Douglas Boyes (University of Oxford). The specimen was identified by Douglas Boyes and snap-frozen on dry ice.

DNA was extracted at the Tree of Life laboratory, Wellcome Sanger Institute. The ilMacNota1 sample was weighed and dissected on dry ice with tissue set aside for Hi-C sequencing. Abdomen tissue was disrupted using a Nippi Powermasher fitted with a BioMasher pestle. Fragment size analysis of 0.01–0.5 ng of DNA was then performed using an Agilent FemtoPulse. High molecular weight (HMW) DNA was extracted using the Qiagen MagAttract HMW DNA extraction kit. Low molecular weight DNA was removed from a 200-ng aliquot of extracted DNA using 0.8X AMpure XP purification kit prior to 10X Chromium sequencing; a minimum of 50 ng DNA was submitted for 10X sequencing. HMW DNA was sheared into an average fragment size between 12–20 kb in a Megaruptor 3 system with speed setting 30. Sheared DNA was purified by solid-phase reversible immobilisation using AMPure PB beads with a 1.8X ratio of beads to sample to remove the shorter fragments and concentrate the DNA sample. The concentration of the sheared and purified DNA was assessed using a Nanodrop spectrophotometer and Qubit Fluorometer and Qubit dsDNA High Sensitivity Assay kit. Fragment size distribution was evaluated by running the sample on the FemtoPulse system.

### Sequencing

Pacific Biosciences HiFi circular consensus and 10X Genomics Chromium read cloud sequencing libraries were constructed according to the manufacturers’ instructions. Sequencing was performed by the Scientific Operations core at the Wellcome Sanger Institute on Pacific Biosciences SEQUEL II (HiFi) and Illumina NovaSeq 6000 (10X) instruments. Hi-C data were generated in the Tree of Life laboratory from head and thorax of ilMacNota1 using the Arima v2 kit and sequenced on a NovaSeq 6000 instrument.

### Genome assembly

Assembly was carried out with Hifiasm ( Cheng *et al.*, 2021); haplotypic duplication was identified and removed with purge_dups ( Guan *et al.*, 2020). One round of polishing was performed by aligning 10X Genomics read data to the assembly with longranger align, calling variants with freebayes ( Garrison & Marth, 2012). The assembly was then scaffolded with Hi-C data ( Rao *et al.*, 2014) using YaHS ( Zhou *et al.*, 2022). The assembly was checked for contamination as described previously ( Howe *et al.*, 2021). Manual curation was performed using HiGlass ( Kerpedjiev *et al.*, 2018) and Pretext. The mitochondrial genome was assembled using MitoHiFi ( Uliano-Silva *et al.*, 2021), which performs annotation using MitoFinder ( Allio *et al.*, 2020). The genome was analysed and BUSCO scores generated within the BlobToolKit environment ( Challis *et al.*, 2020). Table 3 contains a list of all software tool versions used, where appropriate.

**Table 3.** Software tools used.

Software tool	Version	Source
Hifiasm	0.15.3	Cheng *et al.,* 2021
purge_dups	1.2.3	Guan *et al.,* 2020
longranger align	2.2.2	https://support.10xgenomics.com/ genome-exome/software/pipelines/ latest/advanced/other-pipelines
freebayes	1.3.1-17- gaa2ace8	Garrison & Marth, 2012
MitoHiFi	2.0	Uliano-Silva *et al.,* 2021
YaHS	1.0	Zhou *et al.,* 2022
HiGlass	1.11.6	Kerpedjiev *et al.,* 2018
PretextView	0.2.x	https://github.com/wtsi-hpag/ PretextView
BlobToolKit	3.2.6	Challis *et al.,* 2020

### Ethics/compliance issues

The materials that have contributed to this genome note have been supplied by a Darwin Tree of Life Partner. The submission of materials by a Darwin Tree of Life Partner is subject to the Darwin Tree of Life Project Sampling Code of Practice. By agreeing with and signing up to the Sampling Code of Practice, the Darwin Tree of Life Partner agrees they will meet the legal and ethical requirements and standards set out within this document in respect of all samples acquired for, and supplied to, the Darwin Tree of Life Project. Each transfer of samples is further undertaken according to a Research Collaboration Agreement or Material Transfer Agreement entered into by the Darwin Tree of Life Partner, Genome Research Limited (operating as the Wellcome Sanger Institute), and in some circumstances other Darwin Tree of Life collaborators.

## Data availability

European Nucleotide Archive: Macaria notata (peacock moth). Accession number PRJEB48591; https://identifiers.org/ena.embl/PRJEB48591.

The genome sequence is released openly for reuse. The *M. notata* genome sequencing initiative is part of the Darwin Tree of Life (DToL) project. All raw sequence data and the assembly have been deposited in INSDC databases. The genome will be annotated and presented through the Ensembl pipeline at the European Bioinformatics Institute. Raw data and assembly accession identifiers are reported in Table 1.

## Author information

Members of the University of Oxford and Wytham Woods Genome Acquisition Lab are listed here: https://doi.org/10.5281/zenodo.6418202.

Members of the Darwin Tree of Life Barcoding collective are listed here: https://doi.org/10.5281/zenodo.6418156.

Members of the Wellcome Sanger Institute Tree of Life programme are listed here https://doi.org/10.5281/zenodo.6866293.

Members of Wellcome Sanger Institute Scientific Operations: DNA Pipelines collective are listed here: https://doi.org/10.5281/zenodo.5746904.

Members of the Tree of Life Core Informatics collective are listed here: https://doi.org/10.5281/zenodo.6125046.

Members of the Darwin Tree of Life Consortium are listed here: https://doi.org/10.5281/zenodo.6418363.
