# Supplementary material for: The genome sequence of the peacock moth, Macaria notata (Linnaeus, 1758)
Source: Wellcome Open Res. 2022 Sep 12;7:228. [Version 1] doi: 10.12688/wellcomeopenres.18108.1 (PMC10521120; doi:10.12688/wellcomeopenres.18108.1)
Supplement: Peer review history

# Reviewer report: Reviewer response for version 1

**DOI:** 10.21956/wellcomeopenres.20079.r66844

**Published:** 2023-09-12

**Authors:** Jerome H.L Hui

**Affiliations:** 1 Chinese University of Hong Kong, Hong Kong, China

Boyes and colleagues report the genome sequence of peacock moth *Macaria notata *(Linnaeus, 1758). This species can been found in woodland habitat in England and Wales, and there are also reports for its occurrence in Scotland and Ireland. Molecular data of this species are scarce prior to this report, and are mainly confined to COI sequences (plus a few other genes) deposited to the NCBI database. This new genome resource will be very useful for further studies, including but not limited to understanding their evolutionary relationships with other moths, identifying whether there could be cryptic species, or revealing population structure in relations to habitats or geographical locations. 

 This genome resource is excellent from the summary statistics, with high BUSCO numbers, high sequence continuity (contig and scaffold N50), and majority of sequences contained on the 29 pseudochromosomes (plus mitochondrion). To sum up, this is a valuable contribution.

Are sufficient details of methods and materials provided to allow replication by others?

Yes

Is the rationale for creating the dataset(s) clearly described?

Yes

Are the datasets clearly presented in a useable and accessible format?

Yes

Are the protocols appropriate and is the work technically sound?

Yes

Reviewer Expertise:

Genomics, evolution, invertebrates

I confirm that I have read this submission and believe that I have an appropriate level of expertise to confirm that it is of an acceptable scientific standard.

# Reviewer report: Reviewer response for version 1

**DOI:** 10.21956/wellcomeopenres.20079.r54227

**Published:** 2023-02-07

**Authors:** Sam Whiteford

**Affiliations:** 1 University of Liverpool,, Liverpool, UK

The authors report a genome assembly for the moth species *Macaria notata*, representing a mosaic of the two haplotypes present in the sampled individual. The alternate mosaic of haplotypes is also made available. The sequencing technologies used, and assembly methodologies applied are appropriate for generating very high-quality genome representations. Some phenotypic features and ecological background of the organism is given for context. The inclusion of a photograph of the sequenced specimen is nice, and the plots are informative of the data collected and the quality assessments performed. The interactive plots are a nice touch, and useful for taking a closer look at the data, particularly the Hi-C maps. The DNA extraction methodology is described in a good level of detail. The underlying raw data appears to be accessible. The authors provide some standard genome quality assessments such as the BUSCO score and coverage vs GC plots. BUSCO analysis shows a very low number of missing, fragmented and duplicated genes, suggesting that genic regions are complete and an accurate haploid representation has been produced.

 I have a few comments:

 The date that the individual was sampled may be useful metadata to include if available.

 A copy or even rough reference version of the code used for the assembly itself and the subsequent QC steps would be useful. I think the assembly portion is largely reproduceable from the written description, however the exact parameters used for the various programs is important. The manual curation steps are a little opaque (e.g. which underlying scaffold was broken and where & what the resulting scaffolds are called), but I’m sympathetic to the difficulty of publishing a perfectly reproducible genome assembly (especially given the scale of DToL). Also, the availability of multiple intermediate genome assemblies for the sake of complete transparency can create unnecessary confusion.

 One of the chromosomes/pseudomolecules is labelled as the Z chromosome, but I do not see a description of how this assignment was made. This may be important detail for researchers interested in Z evolutionary history. For this reason, I scored the “replication detail” question as partial. Inclusion of the Z chromosome assignment methodology would improve the replicability.

 There are some useful plots presented in the Tree of Life QC webpages that would be nice to include in these genome notes. In particular, the k-mer results from the HiFi data, for a justification of the resulting assembled genome size and to give an estimate of polymorphism, repetitiveness (although the latter points may be beyond the scope of a genome note).

 The snail plot is an aesthetically appealing presentation, but the radial layout combined with the multiple layers might make it tricky to interpret for a general audience

Are sufficient details of methods and materials provided to allow replication by others?

Partly

Is the rationale for creating the dataset(s) clearly described?

Yes

Are the datasets clearly presented in a useable and accessible format?

Yes

Are the protocols appropriate and is the work technically sound?

Yes

Reviewer Expertise:

Lepidoptera genomics/genetics

I confirm that I have read this submission and believe that I have an appropriate level of expertise to confirm that it is of an acceptable scientific standard.
